# A Digital Electrocardiographic System for Assessing Myocardial Electrical Instability: Principles and Applications

**DOI:** 10.17691/stm2020.12.6.02

**Published:** 2020-12-28

**Authors:** A.P. Vorobiev, T.G. Vaykhanskaya, O.P. Melnikova, V.P. Krupenin, V.B. Polyakov, A.V. Frolov

**Affiliations:** Senior Researcher, Laboratory of Medical Information Technologies; Republican Scientific and Practical Center “Cardiology”, Ministry of Health of the Republic of Belarus, 110B Rosa Luxemburg St., Minsk, 220036, Republic of Belarus;; Leading Researcher, Laboratory of Medical Information Technologies; Republican Scientific and Practical Center “Cardiology”, Ministry of Health of the Republic of Belarus, 110B Rosa Luxemburg St., Minsk, 220036, Republic of Belarus;; Senior Researcher, Laboratory of Medical Information Technologies; Republican Scientific and Practical Center “Cardiology”, Ministry of Health of the Republic of Belarus, 110B Rosa Luxemburg St., Minsk, 220036, Republic of Belarus;; System Engineer; Unitary Enterprise “Cardian”, 10, 4^th^ Radiatorny Lane, Minsk, 220093, Republic of Belarus;; Associate Professor, Department of Radio Electronics and Information Security; Perm State University, 15 Bukireva St., Perm, 614990, Russia; Professor, Head of the Laboratory of Medical Information Technologies Republican Scientific and Practical Center “Cardiology”, Ministry of Health of the Republic of Belarus, 110B Rosa Luxemburg St., Minsk, 220036, Republic of Belarus;

**Keywords:** myocardial electrical instability, ECG markers of myocardial instability, predicting adverse cardiovascular events, arrhythmia, sudden cardiac death

## Abstract

**The aim of the study** was to develop an ECG hardware and software system for monitoring electrical instability of the myocardium and to assess the diagnostic and prognostic capabilities of this setup in a cardiology clinic.

**Materials and Methods.:**

The Intecard 7.3 software and hardware system developed in this study makes it possible to measure fluctuations of the ECG amplitude-time parameters using the beat-to-beat mode. Intecard 7.3 evaluates a number of ECG markers that reflect electrical instability of the myocardium. Among them are the fragmented QRS complex, the spatial QRS-T angle, the T-wave alternans, the duration, and dispersion of the QT interval, the turbulence and acceleration/deceleration of the heart rhythm.

Clinical trials of Intecard 7.3 were carried out with 734 patients with ischemic heart disease or cardiomyopathy and 112 healthy individuals.

**Results.:**

Intecard 7.3 reliably identifies fragmented QRS complexes by detecting short spikes of <25 ms in the ascending parts of the Q, R, and S waves. The QRS-T angle is determined from the reference amplitudes of the R and T waves in leads avF, V_2_, V_5_, and V_6_. Digital precision processing of the ECG signal improves its accuracy to microvolts and microseconds.

The software was designed to measure the T-wave amplitude in each of 300–500 cardiobeats; T-wave alternans was estimated by the moving average method. In a typical cardiobeat, the QT dispersion was calculated based on 12 ECG leads. From the sequence of RR intervals, turbulence, and deceleration of the heart rhythm were determined.

During the observation period of 5.0 [2.1; 5.9] years, 90 out of 734 patients (12.3%) experienced adverse cardiovascular events (ACVE). In this period, the myocardial electrical instability was recorded in patients with ACVE more frequently than in those without ACVE. Thus, the frequency of fragmented QRS was 72.2±4.7 vs 16.8±1.5% (p<0.01), the values of the QRS-T angle were 128 [55; 101] vs 80 [53; 121]° (p<0.001), the T-wave alternans — 36.9 [15.5; 62.1] vs 21.9 [10.2; 30.7] μV (p<0.005), the QT interval — 408 [383; 438] vs 376 [351; 400] ms (p<0.001), the QT dispersion — 76 [57; 96] vs 64 [50; 92] ms (p<0.005), respectively. In patients with ACVE, the threshold that triggers pathological rhythm turbulence was higher (>0%) than that in healthy controls (p<0.001); the deceleration of the heart rhythm was reduced from 19.2 [2.2; 38.0] to 8.8 [4.0; 16.8] ms (p<0.05).

A personalized model for ACVE risk stratification has been developed. In this model, the area under the ROC curve was 0.856; sensitivity — 75%; specificity — 78%; predictive accuracy — 77%.

**Conclusion.:**

Using the ECG markers of myocardial electrical instability, the Intecard 7.3 system allows one to predict life-threatening ventricular tachyarrhythmias and sudden cardiac death with an accuracy of 77%. The non-invasiveness, high productivity, and reasonable cost ensure the availability of this predictive technology in all levels of healthcare.

## Introduction

The recent progress in electrocardiography (ECG) has become possible due to the development of precision digital signal processing. New diagnostic qualities of ECG were enabled by using the dispersion, high-frequency, and phase analysis. A visual portrait of the myocardium with its ischemic zones was obtained [1]; high-frequency ECG components provided the clues to diagnosing stenosis in the right coronary artery [2]; the phase analysis of ECG was found more sensitive than the temporal one [3]. These and similar developments demonstrate the inexhaustible diagnostic potential of the ECG analysis.

Since the beginning of the 2000s, the beat-to-beat analysis of fluctuations of the ECG amplitude-time parameters has been increasingly used. A close relationship between the dispersion of fluctuations and the risk of life-threatening arrhythmias and mortality was found by Lampert [4]; she also introduced the term “irritated heart” into the literature. Later, this condition was transformed into the “electrical instability of the myocardium”. Fragmentation of the QRS complex and the spatial QRS-T angle are considered the major ECG markers of electrical instability in the depolarization phase. Heart instability in the repolarization phase is reflected by the T-wave alternans as well as the duration and dispersion of the QT interval. Turbulence and decelerating/accelerating heart rate were proposed as ECG markers of the heart autonomic control dysfunction. For all markers of myocardial electrical instability, international consensus on the measurement standards and clinical interpretation has been adopted [5–10]. Taken together, these markers enhance the diagnostic and prognostic capabilities of traditional ECG analysis. However, the lack of specialized software and hardware for this analysis impedes their use in clinical practice.

**The aim of the study** was to develop an ECG hardware and software system for monitoring electrical instability of the myocardium and to assess the diagnostic and prognostic capabilities of this setup in a cardiology clinic.

## Materials and Methods

The study is based on a recently developed original hardware and software system named Intecard 7.3, which applies precision digital processing of the ECG data to evaluating the markers of myocardial electrical instability (MEI). The system was created on the platform of a 12-channel digital ECG communicator, a computer, and a laser printer. The amplitude range of the recorded ECG signals was from 0.03 to 5 mV, the input impedance >10 MΩ, the common mode rejection ratio — 110 dB, the time constant >3.2 s, the sampling rate — 1000 Hz/channel, the number of bits — 24, and the output interface — USB 2.0. The complex is equipped with a set of adaptive digital filters for power, muscle, and respiratory interferences; the filters do not distort the original ECG shape.

We followed the internationally accepted criteria to detect MEI in the depolarization phase, namely: by fragmentation of QRS complexes in two or more ECG leads and/or by a QRS-T angle of >105°. In the repolarization phase, MEI was identified at T-wave alternans >47 μV and/or QT dispersion >70 ms. Dysfunction of the autonomic control was diagnosed by pathological heart rhythm turbulence (onset >0%, slope <15 ms/RR) and/or deceleration of the heart rhythm <4.5 ms.

To assess the diagnostic and prognostic capabilities of the Intecard 7.3 system, 846 people were examined; of those, 734 patients with ischemic heart disease or cardiomyopathy (the average age 50±15 years) and 112 healthy individuals (31±14 years). The adverse cardiovascular events (ACVE) followed in these patients included sustained ventricular tachycardia, ventricular fibrillation, successful cardiopulmonary resuscitation, discharges of implanted devices, and signs of sudden cardiac death. The observation period in these patients was 5.0 [2.1; 5.9] years. The study was conducted in accordance with the Declaration of Helsinki and approved by the Ethics Committee of the Republican Scientific and Practical Center “Cardiology” (Republic of Belarus). Informed consent was obtained from each patient.

**Statistical analysis.** The data was processed using the Statistica 10.0 (StatSoft) and SPSS Statistics 23.0 (IBM) application packages. The results are presented as M±SD or Me [Q1; Q3] depending on the type of data distribution. The normal distribution was tested using the Shapiro–Wilk test. We also used the method of categorical regression and determined the relative risk with a 95% confidence interval. Differences between the groups were analyzed for significance using the Student’s t-test, Pearson’s χ^2^ test, or Mann–Whitney U-test, depending on the type of data distribution. The quality of the predictive model was assessed by the area under the ROC curve and the predictive accuracy. The critical value of statistical significance when testing the null hypotheses was taken equal to 0.05.

## Results

**Figure 1:**
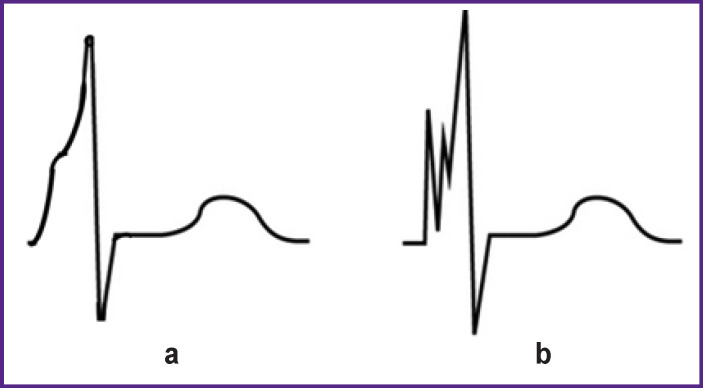
Fragmented QRS complex with spikes on the ascending part of the R-wave: (a) ECG recording with the upper passband frequency of 40 Hz; (b) digital ECG recording with a sampling frequency of 1000 Hz and an upper passband frequency of >100 Hz

By means of the ECG precision digital processing, we were able to increase the accuracy of the Intecard 7.3 analysis up to several microvolts and microseconds; this result reflects the evolutionary transition to the 4^th^ generation of electrocardiography. Here, we demonstrate the way to reliably identify fragmented QRS (frQRS) complexes — one of the markers of myocardial scarring, ischemia, and fibrosis. Around these zones, boundary layers with slow excitation/conduction rates are formed. In the ECG, these changes appear as short spikes (of less than 25 ms) on the Q, R, or S waves. A regular ECG record is able to detect the splitting of the R-wave apex, but it fails to detect spikes on the ascending parts of the Q, R, or S waves. With the help of Intecard 7.3 at a sampling rate of 1000 Hz, the system accumulates a sufficient number of ECG readings, which allows identifying frQRS spikes by using high order derivatives. This can be seen by comparing the ECG recorded with the upper passband frequency of 40 Hz (Figure 1 (a)) and the same ECG recorded with a sampling frequency of 1000 Hz and the upper passband frequency of >100 Hz (Figure 1 (b)). The second ECG record clearly shows frQRS spikes missed in the first record.

In heterogeneous myocardial tissue, the gradient of the excitation wave is defragmented, which leads to a prolongation of the depolarization phase and a delay in the repolarization phase. The degree of heterogeneity of the myocardial matrix is proportional to the angle between the QRS and T vectors. Orthogonal XYZ leads are usually used to measure the QRS-T angle. In this study though, we assessed the QRS-T angle using a technique adapted to 12 ECG leads [11], i.e., leads avF, V_2_, V_5_, and V_6_, in which the native R and T amplitudes were converted to the respective reference points:


QRSrep = R−abs(maxQ or S), Trep = T(+)−absT(−),

where *QRSrep* — reference value of QRS; *R* — R amplitude; *abs* — absolute value; *max* — the maximum Q or S waves; *Trep* — reference value of T-wave; *T*(*+*), *T*(–) — the largest positive and negative amplitudes of T-wave.

The Intecard 7.3 software accumulates 300–500 cardiobeats and measures the T-wave amplitudes in each of them. Using the moving average method, the averaged values of T in even and odd cardiobeats are determined. The difference between these values is a measure of T-wave alternans (in microvolts) [7]. In addition, in a typical cardiobeat, dispersion of the QT interval is determined by the difference between the maximum and minimum QT values in 12 ECG leads [8].

Ventricular extrasystoles are detected by changes in the heart rhythm turbulence: TO — onset and TS — slope [10]. In addition, the Intecard 7.3 software provides for defragmentation of the RR intervals sequence into sections of acceleration and deceleration. The method of synchronous detection measures the acceleration and deceleration of the heart rate [10].

In clinical trials of the Intecard 7.3 system, 644 out of 734 patients (87.7%) had no ACVE, and 90 patients (12.3%) did have ACVE. In Table 1, the data on ECG markers of MEI in healthy subjects (group 1), patients without (group 2) and with ACVE (group 3) are shown.

**Table 1:** Values of ECG markers of myocardial electrical instability in healthy individuals and cardiac patients (Me [Q1; Q3])

ECG markers	Group 1 — control (n=112)	Group 2 — patients without ACVE (n=644)	Group 3 — patients with ACVE (n=90)	p
Mean age (years) (M±SD)	31±14	52±17	47±13	p_1, 2_<0.001
				p_2, 3_>0.10
Left ventricular ejection fraction (%)	64 [61; 69]	36 [27; 54]	34 [23; 58]	p_1, 2_<0.001
				p_1, 3_<0.001
				p_2, 3_>0.10
QRS fragmentation (%) (M±SD)	13.4±3.2	16.8±1.5	72.2±4.7	p_1, 2_>0.05
				p_1, 3_<0.01
				p_2, 3_<0.01
QRS-T angle (degrees)	69 [35; 102]	80 [53; 121]	128 [101; 155]	p_2, 3_<0.001
T-wave alternans (μV)	15.7 [8.0; 29.3]	21.9 [10.2; 30.7]	36.9 [15.5; 62.1]	p_2, 3_<0.005
QT duration (ms)	367 [339; 394]	376 [351; 400]	408 [383; 438]	p_2, 3_<0.001
QT dispersion (ms)	57.6 [34; 85]	64.5 [50; 92]	76 [57; 96]	p_2, 3_<0.005
HRT, onset (%)	–10.4 [–15.3; 0.7]	1.0 [–6.1; 10.5]	2.0 [–4.4; 5.7]	p_1, 2_<0.005
HRT, slope (ms/RR)	35 [17; 62]	31 [12; 57]	20 [12; 46]	p_2, 3_>0.10
Heart rhythm deceleration (ms)	19.2 [2.2; 38.0]	10.8 [5.8; 24.5]	8.8 [4.0; 16.8]	p_1, 2_<0.05

Here: HRT — heart rhythm turbulence.

As per Table 1, the values of the MEI parameters practically do not differ between groups 1 and 2; on the contrary, there were statistically significant differences in these values between patients of groups 2 and 3. Thus, in patients of group 3, the marker values significantly increased: in the depolarization phase, these were the frequency of detection of frQRS (p<0.01) and the QRS-T angle (p<0.001); in the repolarization phase, those were the T-wave alternans as well as the duration and dispersion of the QT interval (p<0.005). The markers of autonomic nervous regulation — the turbulence and slowing of the heart rate — did not significantly differ between the groups.

## Discussion

Clinical trials of the Intecard 7.3 system have demonstrated its ability to extract useful diagnostic and prognostic information from ECG records. For example, the traditional Q-wave marker is leveled or even disappears after myocardial infarction. In contrast, the frQRS complex is stable over time and therefore its sensitivity is 2.3 times higher [12]. In addition, frQRS is helpful in localizing stenosis in coronary arteries. Thus, the appearance of frQRS in chest leads V_1_ to V_6_ indicates stenosis in the left coronary artery, and the presence of frQRS in inferior leads II, III, and avF points to stenosis in the right artery [13].

According to the present results, only in patients of group 3 (those with ACVE), the threshold value of the QRS-T angle (105°) is exceeded; that indicates a significant disturbance of the synergy between excitation and relaxation of the myocardium.

In patients of group 3 (ACVE), MEI in the repolarization phase was expressed by a twofold increase in the T-wave alternans and an 18% increase in the QT dispersion as compare with group 2. It is no coincidence that the repolarization phase is considered the most vulnerable one during arrhythmogenesis. In this group, the indicators of turbulence and deceleration of the heart rhythm showed statistically significant changes in comparison with the control group, which was indicative of deterioration in the autonomic nervous regulation.

The significant differences in MEI markers between patients without and with ACVE were taken as the basis for our prognostic model. These markers included frQRS, QRS-T angle, T-wave alternans, and QT interval, which had the largest areas under the ROC curves. The “importance factors” reflecting the contribution of each marker in the likelihood of ACVE were determined. Table 2 shows the relative risk values and regression coefficients of the risk stratification model developed using the categorical regression method with optimal scaling.

**Table 2:** Relative risk and coefficients of the risk stratification model based on ECG markers of myocardial electrical instability

Predictive ECG markers	Relative risk at 95% CI	Bootstrap/ mean square error	Importance factor	Fisher’s F test	p
QRS fragmentation	4.31 (3.48–5.34)	0.322/0.060	0.462	28.805	0.000
QRS-T angle	2.15 (1.81–2.56)	0.246/0.050	0.268	23.843	0.000
T-wave alternans	4.12 (2.45–6.93)	0.164/0.067	0.153	5.967	0.003
QT interval	2.03 (1.34–2.66)	0.132/0.050	0.117	7.056	0.000

The model with normalized coefficients looks as follows:


$$F(x)=a_{1}\cdot x_{1}+a_{2}\cdot x_{2}+a_{3}\cdot x_{3}+a_{4}\cdot x_{4},$$

where *F*(*x*) is the integral index of MEI; *x*_1_=1 if a frQRS is detected, otherwise *x*_1_=0; *x*_2_=1 if QRS-T >105°, otherwise *x*_2_=0; *x*_3_=1 if T-wave alternans >47 μV, otherwise *x*_3_=0; *x*_4_=1 if the QT interval >395 ms, otherwise *x*_4_*=*0; *a*_1_=46; *a*_2_=27; *a*_3_=15; and *a*_4_=12.

If the *F*(*x*) value ranges from 0 to 25, then the risk of ACVE is low; from 25 to 50 — moderate; at 50–75 — high and at >75 — critical.

**Figure 2:**
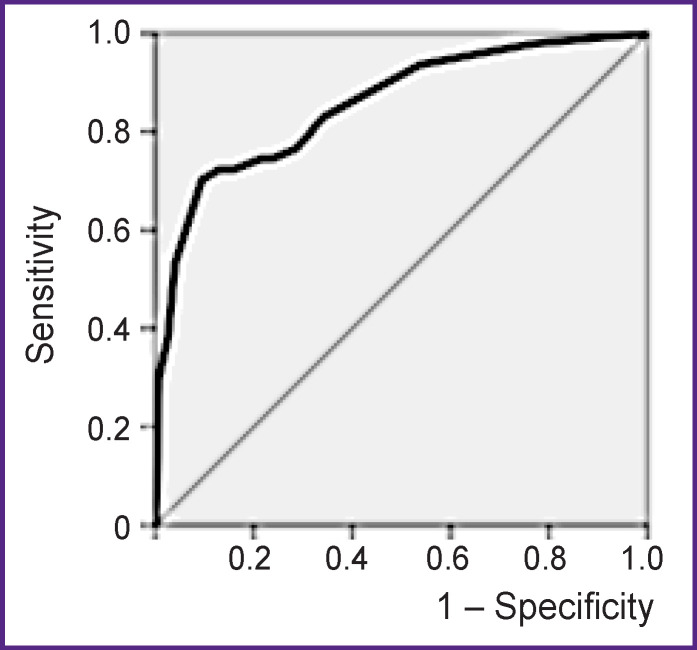
ROC curve of the risk stratification model based on ECG markers of myocardial electrical instability The area under the ROC curve — 0.856; sensitivity — 75%; specificity — 78%; predictive accuracy — 77%

The quality of the risk stratification model was assessed by the area under the ROC curve (AUC). In this case, AUC=0.856, sensitivity — 75%, specificity — 78%, predictive accuracy — 77%, which indicates a good quality of the prediction (Figure 2). The predictive accuracy of 77% is considered acceptable.

The well-known risk stratification models Euroscore II, Timi, Seattle Risk Score, and others are based on traditional population risk factors. They are good in predicting hospital mortality (AUC — 0.73–0.87), but for long-term prediction their accuracy is low [14]. In contrast, the prediction technology developed in this study is personalized, as it is based on the myocardium bioelectrical activity of an individual patient. Using the MEI index, it is possible to identify individuals with a high risk of sudden cardiac death and recommend the implantation of rhythm-supporting devices.

Limitations of the study include its monocentric affiliation and also the absence of standards for using MEI markers in clinical practice of the Russian Federation and the Republic of Belarus.

## Conclusion

The ECG hardware and software system Intecard 7.3 allows for predicting life-threatening ventricular tachyarrhythmias and sudden cardiac deaths. The setup involves precision digital processing of ECG records and predicts myocardial electrical instability with an accuracy of 77%.

The non-invasiveness, high productivity, and reasonable cost ensure the availability of this predictive technology at all levels of healthcare, including primary care institutions.
